# Predictors of latent tuberculosis treatment initiation and completion at a U.S. public health clinic: a prospective cohort study

**DOI:** 10.1186/1471-2458-12-468

**Published:** 2012-06-21

**Authors:** Neela D Goswami, Lara Beth Gadkowski, Carla Piedrahita, Deborah Bissette, Marshall Alex Ahearn, Michela LM Blain, Truls Østbye, Jussi Saukkonen, Jason E Stout

**Affiliations:** 1 Duke University Medical Center, Durham, NC, USA; 2 Wake County Human Services, Raleigh, NC, USA; 3 Duke University, Durham, NC, USA; 4 Boston University School of Medicine, Boston, MA, USA

**Keywords:** Adherence, LTBI, Compliance, Attitude, Geographic

## Abstract

**Background:**

Treatment of latent tuberculosis infection (LTBI) is a key component in U.S. tuberculosis control, assisted by recent improvements in LTBI diagnostics and therapeutic regimens. Effectiveness of LTBI therapy, however, is limited by patients’ willingness to both initiate and complete treatment. We aimed to evaluate the demographic, medical, behavioral, attitude-based, and geographic factors associated with LTBI treatment initiation and completion of persons presenting with LTBI to a public health tuberculosis clinic.

**Methods:**

Data for this prospective cohort study were collected from structured patient interviews, self-administered questionnaires, clinic intake forms, and U.S. census data. All adults (>17 years) who met CDC guidelines for LTBI treatment between January 11, 2008 and May 6, 2009 at Wake County Health and Human Services Tuberculosis Clinic in Raleigh, North Carolina were included in the study. In addition to traditional social and behavioral factors, a three-level medical risk variable (low, moderate, high), based on risk factors for both progression to and transmission of active tuberculosis, was included for analysis. Clinic distance and neighborhood poverty level, based on percent residents living below poverty level in a person’s zip code, were also analyzed. Variables with a significance level <0.10 by univariate analysis were included in log binomial models with backward elimination. Models were used to estimate risk ratios for two primary outcomes: (1) LTBI therapy initiation (picking up one month’s medication) and (2) therapy completion (picking up nine months INH therapy or four months rifampin monthly).

**Results:**

496 persons completed medical interviews and questionnaires addressing social factors and attitudes toward LTBI treatment. 26% persons initiated LTBI therapy and 53% of those initiating completed therapy. Treatment initiation predictors included: a non-employment reason for screening (RR 1.6, 95% CI 1.0-2.5), close contact to an infectious TB case (RR 2.5, 95% CI 1.8-3.6), regular primary care(RR 1.4, 95% CI 1.0-2.0), and history of incarceration (RR 1.7, 95% CI 1.0-2.8). Persons in the “high” risk category for progression/transmission of TB disease had higher likelihood of treatment initiation (p < 0.01), but not completion, than those with lower risk.

**Conclusions:**

Investment in social support and access to regular primary care may lead to increased LTBI therapy adherence in high-risk populations.

## Background

Latent tuberculosis infection (LTBI) treatment is a key strategy to reduce TB disease incidence in industrialized countries. Despite recent advances in LTBI diagnostics and up to 90 percent efficacy of first-line therapy for LTBI if taken properly, the proportion of patients who complete prescribed therapy remains low, ranging from 50-65% [1-3]. Firstly, LTBI therapy initiation requires patient motivation and understanding of the diagnosis, adequate communication between health department staff and the patient, and availability of transportation to pick up initial medications; barriers can arise to any of these components. Therapy completion is a second, independent step with potentially different and additional barriers at each visit, such as increased fear of medication side effects, logistical issues, or negative attitudes towards treatment that emerge over time.

Suboptimal compliance with traditional TB preventative therapy has been attributed to various factors, including duration of therapy, concerns about drug toxicity, and pill burden [4]. Studies comparing four months of rifampin or two months of rifampin and pyrazinamide to the traditional nine months of isoniazid (INH) note improved adherence to the shorter regimens [5-7], but increased hepatotoxicity with rifampin-pyrazinamide led to withdrawal of the regimen from CDC recommendations [8]. Studies evaluating three months of rifampin plus INH versus six months of INH, have noted no significant difference in completion rates, possibly secondary to the increased pill burden of a two-drug regimen [9]. A study of three months directly-observed therapy with rifapentine and INH versus self-administered nine months INH was also recently completed, with adherence data forthcoming [10].

Aside from factors relating to therapy, other predictors of medication adherence have been less prospectively studied. Factors that have been consistently associated with treatment completion in retrospective studies include non-U.S. birthplace [11-13] and recent exposure to an active tuberculosis case [4,14]. Age and other demographics have been less consistently associated with treatment adherence across studies, with some suggesting older age and others suggesting younger age correlating with therapy adherence [11,13-16]. Certain patient attitudes and social factors have also been correlated with LTBI therapy non-completion, including perception of a low risk of progression to active TB without LTBI treatment, dislike of venipuncture [16], fear about side effects [11,15], lower educational level [4,17], and unstable housing [4,11].

As identifying LTBI, initiating therapy, and adherence to LTBI therapy serve as the cornerstones of U.S. TB control efforts, prospective studies evaluating predictive factors in treatment completion will not only help modernize and improve treatment strategies, but also allow more educated and tailored interventions in cases of noncompliance. The aim of this study was to understand barriers to LTBI treatment initiation and completion by determining demographic, medical, behavioral, attitude-based, and geographic factors potentially associated with therapy adherence in LTBI-infected patients recommended for treatment by the Wake County TB clinic in Raleigh, North Carolina. Based on anecdotal experience in the clinic, we hypothesized that those persons at highest risk of progressing to active TB disease and spreading TB infection to others, as well as those living in poorer geographical areas, would be less likely to initiate and complete therapy.

## Methods

### Study setting and patient recruitment

All adults (>17 years) who met CDC guidelines for LTBI treatment between January 11, 2008 and May 6, 2009 at Wake County Tuberculosis Clinic in Raleigh, North Carolina were included in the study. Written informed consent was waived by the Duke Institutional Review Board, as this was considered an evaluation of a public benefit program, and thus eligible for such a waiver under the United States Code of Federal Regulations part 45 46.116(c)(1). The study was also approved by the Wake County Human Rights-Consumer Affairs and Human Research Committee.

### Data collection

On entry to the TB clinic, a routine medical interview was conducted, followed by a self-administered multiple choice questionnaire addressing social factors and attitudes toward LTBI treatment (Table 1). The questionnaire was developed based on the results of a focus group study assessing TB-related knowledge, attitudes, and behaviors among key populations in the region [18]. The questionnaire was validated by administering it to a pilot population of individuals taking isoniazid therapy at baseline and one month later; in this group test-retest response concordance was >0.75.

**Table 1:** Latent TB infection therapy initiation and completion based on responses to survey questions

**Variable**	**Treatment Initiation (%)**	**P-value**	**Treatment Completion (%)**	P-value^*^
Length of time at current residence				
Less than 1 yr	20	0.01	52	0.8
Greater or equal to 1 year	31		55	
Planned future time at current residence				
Less than 1 yr	19	0.04	46	0.3
Greater or equal to 1 year	28		58	
No school	50	0.01	0	0.5
Elementary school	41		61	
High school/GED	30		49	
College	18		64	
Graduate school	23		53	
Cohabitance with any family members				
Yes	27	0.6	57	0.3
No	24		47	
Previous daily pill for at least 6 months				
Yes	29	0.2	49	0.3
No	24		59	
Regular primary care				
Yes	37	<0.01	52	0.5
No	19		59	
Easy access to health department				
Yes	28	0.1	54	0.8
No	19		57	
Plan to tell family/friends about positive skin test				
Yes	25		59	
No	35	0.1	30	0.02
Belief in getting sick from TB without medicine				
Yes	34	<0.01	53	0.6
No	15		58	
Fear of adverse effects from medicine				
Yes	24	0.3	55	0.8
No	28		52	
Belief in medicine efficacy				
Yes	28	0.3	55	0.6
No	21		46	
Belief in cure for TB				
Yes	26	0.9	55	0.7
No	27		60	
Fear of phlebotomy				
Yes	25	0.8	50	0.7
No	26		56	

^*^P-values represent differences in initiation/completion among responses (missing responses not included).

### Provision of treatment

All LTBI medications were provided free of charge by the health department from an on-site pharmacy, which recorded all medication pickups in a computerized system.

### Statistical methods

Primary endpoints were 1) LTBI therapy initiation, defined as picking up at least one month of medication, and 2) therapy completion, defined as picking up nine months of INH therapy within a 12-month period or four months of rifampin within a six-month period. Univariate analysis was performed with survey responses, demographics, and medical risk data, using chi square test or Fischer’s exact test for categorical variables as appropriate. For multi-level categorical variables, pairwise testing was performed with chi square Univariate analysis was also performed to examine the relationship between the risk that an individual would transmit TB in the future and the likelihood of a) initiating and b) completing LTBI treatment. Subjects were placed in three risk categories, which were based on a combination of medical risk (risk of progression to active TB) and social risk (transmission risk due to suboptimal healthcare utilization). Subjects with history of transplant, HIV, silicosis, intravenous drug use, close contact to an infectious TB case, or with evidence of old tuberculosis on chest radiograph were placed in the “High risk” category. Persons with diabetes, end-stage renal disease, history of gastrectomy, homelessness, or being underweight were placed in the “Moderate risk” category, and all others were considered “Low risk.” Backward elimination was used to arrive at a final log binomial model consisting of independent variables significantly associated with completion of LTBI therapy at p < 0.10 by univariate analysis plus any significant confounders. Significant confounding was defined as a change in the risk ratio for a second variable of interest of >10% when the confounder was included (vs. not included) in the model.

### Geospatial analysis

Residences of study participants were geocoded and mapped using ArcMap 9.3 GIS software. The point density analyst tool was used to calculate distances between each residence address and the Wake County Health Department. Mean distance from the health department was compared between those who initiated and completed therapy and those who did not, using the student’s t-test. Residence zip codes were linked to percentage of area residents living below the poverty line in each zip code, based on Census 2000 data. Zip codes with less than 5% of the population living below the poverty line were categorized as “Low poverty,” those with 5-10% below the poverty line “Moderate poverty” and those with greater than 10% below the poverty line “High poverty.” Univariate relationships between these three categories and LTBI therapy initiation and completion were assessed.

## Results

### Patient characteristics

The 496 participants were predominantly foreign-born (65%) and racial/ethnic minorities (87%), with mean age 39.1 + 12.3 years (Table 2). Of the 496, 130 (26%) persons initiated LTBI therapy and 70 (54% of those initiating) completed therapy. Of those who were foreign-born, the most frequent regions of origin were Africa (19%) and Latin America (20%). 61% of persons included in the study were referred after a tuberculin skin test (TST) was performed as part of employment screening, and 19% received a TST as part of a contact investigation. The overall cohort included a number of former or current smokers (32%), drug users (9%), and persons with history of incarceration (14%) or homelessness (11%).

**Table 2:** Demographics, Risk Factors and Behaviors among Study Population with Latent TB Infection

**Variable**	**N (%) or**	**N (%) or**	**P value**	**N (%) or**	**P value**^€^
	**Mean (SD)**	**Mean (SD)**		**Mean (SD)**	
	**Total Population**	**Who Initiated Therapy**		**Who Completed Therapy**	
Race/ethnicity					
Asian/Pacific					
Islander	70 (14)	16 (23)	0.2	12 (75)	0.1
Non-Hispanic White	64 (13)	10 (16)		3 (30)	
Non-Hispanic Black	254 (51)	67 (26)		39 (58)	
Hispanic	92 (19)	28 (30)		12 (43)	
Other/unknown	16 (3)				
Gender					
Male	236 (48)	67 (28)	0.3	39 (58)	0.3
Female	260 (52)	63 (24)		31 (49)	
Mean age	39.1 (12.3)	42.1 (38.0)	<0.01	42.8 (41.2)	0.5
US-born	169 (34)	57 (34)	0.01	26 (46)	0.07
Region of birth					
Developed world (US, Canada,					
Western Europe, Japan)^*^	175 (35)	57 (33)	0.01	26 (46)	0.03
Africa	94 (19)	17 (18)	0.23	13 (77)	0.04
Asia	65 (13)	16 (25)	0.07	12 (75)	1
Eastern Europe	13 (3)	1 (8)	0.87	0 (0)	0.8
Latin America	98 (20)	31 (32)	0.06	15 (48)	1
Middle East	19 (4)	2 (11)	0.12	1 (50)	1
Other/unknown	32 (7)	6 (19)		3 (50)	
Reason for skin testing					
Employment screening ^*^	304 (61)	50 (17)	<0.01	25 (50)	0.3
Contact to TB case	94 (19)	50 (53)	<0.01	30 (60)	1
Non-employment reason	98 (20)	30 (31)		15 (50)	
Mean distance from health department (miles)		7.9 (9.5)	0.4	8.0 (7.7)	0.9
Neighborhood poverty level					
Low poverty	74 (20)	16 (22)	0.6	13 (81)	0.08
Moderate poverty	155 (41)	39 (25)		19 (49)	
High poverty	149 (39)	42 (28)		22 (42)	
Alcohol use					
None	316 (64)	82 (26)	0.7	41 (50)	0.3
<1 drink/day	106 (21)	31 (29)		22 (71)	
1-2 drinks/day	23 (5)	5 (22)		2 (40)	
3+ drinks/day	23 (5)	4 (17)		2 (50)	
Binge	15 (3)	5 (33)		2 (40)	
Smoking					
None	322 (65)	84 (26)	0.7	51 (61)	0.04
Former	59 (12)	18 (31)		10 (56)	
Current	100 (20)	25 (25)		8 (32)	
Crack cocaine use	34 (7)	12 (35)	0.2	4 (33)	0.2
Diabetes	28 (6)	11 (39)	0.1	7 (64)	0.5
ESRD	3 (1)	3 (100)	0.02	1 (33)	0.6
Gastrectomy	6 (1)	3 (50)	0.2	3 (100)	0.3
Heroin use	8 (2)	2 (25)	1	0 (0)	0.2
Homeless	53 (11)	18 (34)	0.2	8 (44)	0.4
IV drug use	12 (2)	3 (25)	1	0 (0)	0.1
Immunosuppressed	7 (1)	5 (71)	0.02	1 (20)	0.2
Prior incarceration	67 (14)	24 (36)	0.06	9 (38)	0.07
Long term care facility	71 (14)	12 (17)	0.05	8 (67)	0.4
Evidence of old TB on CXR	11 (2)	1 (9)	0.3	0 (0)	0.4
History of organ or bone marrow transplantation	1 (0)	0 (0)	1	0 (0)	1
Underweight	9 (2)	2 (22)	1	1 (50)	1
HIV	6 (1)	4 (67)	0.03	0 (0)	0.05

*Used as reference category for 2-group comparisons to calculate p-value.

^€^ P-values represent differences in proportions initiating/completing treatment among responses (not including missing responses).

### LTBI treatment initiation

In the univariate analysis, factors significantly associated with treatment initiation (P < 0.10) included older age, African birthplace, close contact with an infectious TB case, non-employment reason for TST, end-stage renal disease (ESRD), immunosuppressed status, HIV infection, previous incarceration, residence in current home for greater than one year, plans to remain at same home greater than one year, lower educational level, having a regular physician, and fear of getting sick with tuberculosis (Tables 1 and 2). In multivariable analysis, close contact with a TB case, non-employment reason for screening, lower educational level, having a regular physician, fear of getting sick with TB, and prior incarceration were independently associated with treatment initiation (Table 3). Since there were few (n < 20) persons with specific medical risk factors (ESRD, immunosuppressed status, HIV), medical and social risk were collapsed into three risk strata as described in the Methods section. More persons at “high risk” of progressing to and/or transmitting active TB initiated LTBI therapy (54/120, 45%) than those at “moderate risk” (24/69, 35%) or “low risk” (52/307, 17%)(P < 0.01).

**Table 3:** Multivariable model (log binomial) predicting initiation and completion of treatment by backward elimination

**Category**	**Factor**	**RR**	**95% CI**
Treatment Initiation	Close contact to a TB case	2.5	1.8-3.6
	Non-employment reason for screening	1.6	1.0-2.5
	Lower educational level	1.3	1.1-1.6
	Having a regular physician	1.4	1.0-2.0
	Fear of getting sick with TB without medicine	1.7	1.2-2.6
	Prior incarceration	1.7	1.1-2.8
Treatment Completion	Plan to tell friends or family about LTBI diagnosis	2.0	1.0-3.9

### LTBI treatment completion

Among those initiating treatment, factors associated with treatment completion (P < 0.10) included African or Asian birthplace, absence of tobacco history, prior incarceration, and plan to tell friends/family about positive TST. In multivariable analysis, planning to tell friends/family about positive TST was independently and significantly associated with treatment completion (RR 2.0, 95% CI 1.0-3.9). The proportion of persons at high risk of progressing to and/or transmitting active TB completing therapy was not significantly different from those at moderate or low risk (p = 0.6).

**Figure 1:**
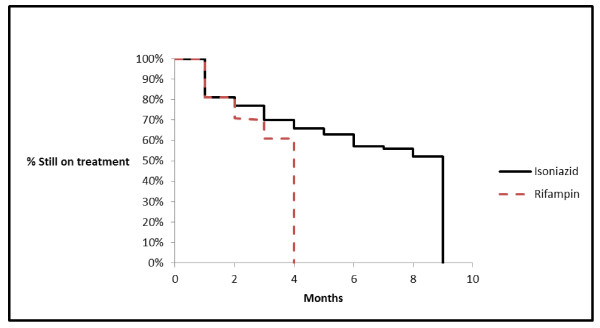
**Adherence to latent tuberculosis infection by therapy, Wake County 2008–2009.** In this study, of persons initiating therapy, 31 were prescribed rifampin, with a 61% completion rate, and 99 persons were prescribed INH, with a 52% nine-month completion rate (p = 0.3). At least six months of INH was completed by 62/99 (63%) persons. Compliance with both therapies declined over time.

Of those persons initiating therapy, 31 were prescribed rifampin, with a 61% completion rate, and 99 persons were prescribed INH, with a 52% nine-month completion rate (p = 0.3). At least six months of INH was completed by 62/99 (63%) persons. Seven persons were switched from isoniazid to rifampin, and four of these completed a full course of rifampin. One person was switched from rifampin to isoniazid and that individual completed a full course of isoniazid. Compliance with both therapies declined over time (Figure 1).

### Geospatial analysis

In geographic analysis, 391 residential addresses were mapped with ArcGIS software. Of the 130 who initiated therapy, 99 participants had addresses that could be mapped to a physical location in North Carolina. Distance from the health department was not significantly different between addresses of persons who initiated LTBI therapy and those who did not initiate therapy (p = 0.4). Similarly, treatment completion was not significantly associated with distance from the health department (p = 0.9). Of all participants, 378 participants had zip codes with poverty data available from the 2000 U.S. census. Treatment initiation did not vary significantly by whether a participant lived in a high, moderate, or low-poverty level area (p = 0.6). Of those who initiated therapy, there was a trend toward greater completion of therapy by persons living in a low-poverty area (81% of those initiating completed therapy), compared to those living in a moderate (49% completed) or high poverty area (42%), at p = 0.08.

## Discussion

Previous authors have evaluated demographic and medication-based predictors of LTBI treatment non-completion, but few, if any, have prospectively evaluated a combination of pre-treatment attitudes, behaviors, geographic, and medical risk factors as they relate to both initiating and completing therapy.

Furthermore, while several studies have evaluated medication completion in a cohort of patients already initiated on therapy [2,4,14,16], one of the most interesting observations in our study was the fact that initiating, rather than completing, treatment was the major challenge in the LTBI program. While about half (55%) of persons initiating therapy completed, only about a quarter (26%) even initiated therapy by picking up the first month’s supply of pills. The initiation rate in our study is notably less than the 91% LTBI therapy initiation rate seen in a cohort of patients presenting to TB clinic at Boston Medical Center [12], 58% initiation rate in a population of health care workers in Toronto [19], and 67% initiation rate in a cohort of HIV-infected patients in Spain [20]. Differences in initiation rates between our study and others may be attributable to different clinic populations, healthcare systems, and the fact that our study, as a program evaluation, included all persons prescribed LTBI treatment without selection bias. For comparison, among all contact investigations conducted in the U.S. in 2006, 72% of those infected with latent TB started treatment [21]. Among contacts in North Carolina in 2009, 67% of those infected started treatment (Kitty Herrin, PhD, personal communication), consistent with our data that close contacts to an infectious TB case are 2.5 times as likely to start therapy than non-contacts.

This study also demonstrated that those with prior incarceration were also more likely to initiate therapy, likely because LTBI treatment was started in a jail or prison setting, although this data was not specifically documented. Routine access to medical care with a regular physician also increased likelihood of therapy initiation, suggesting that continuity of primary care can improve TB control efforts.

Contrary to our hypothesis, persons we classified as higher risk of progression to active TB were more likely to initiate LTBI treatment than persons at lower risk. Unfortunately, even in these high-risk groups, though, a relatively low proportion of participants with LTBI initiated treatment. The likelihood that LTBI treatment would be completed once initiated had no relationship to risk group. Completion was associated with the plan to disclose LTBI diagnosis with friends or family, suggesting that invitation of support members to clinic visits or to otherwise engage in a patient’s care may improve completion rates. Other novel and cost-efficient interventions need to be studied to improve both initiation and completion of LTBI treatment. In a California study of LTBI treatment in drug users, therapy completion rate was 53% when active outreach was used with a financial incentive of five dollars per visit, compared to 4% when active outreach was used alone (p < 0.0001) [22]. A nonmonetary incentive such as a nutritional supplement has also been associated with increased LTBI therapy completion [23]. Directly observed therapy has had less success in increasing treatment completion [24], and educational interventions have had variable impact on adherence.

The impact of a case of active TB may differ based upon where the case occurs. A case in a relatively high-poverty area, further from the health department, may take longer to detect because of financial and logistical barriers to seeking TB-related healthcare [15,17]. While distance from the health department and the economic status of the patient’s neighborhood were not related to the probability of initiating LTBI treatment, of those who initiated therapy, there was a trend toward greater completion of therapy by persons living in low-poverty areas than persons living in higher poverty areas. The higher completion in lower-poverty areas may be due to ease of access to healthcare, local social factors, or other unmeasured factors. Also, while distance to the health department from a participant’s residential address was not significantly related to the probability of initiating or completing LTBI treatment, the distance measures used in our study are only rough approximations of the time required to get to the health department.

In terms of study limitations, results may be less generalizable given this was a single-clinic cohort. Furthermore, employment screening, a less targeted approach, was a frequent reason for TST placement and medication prescription in this clinic; this type of screening was discontinued in part as a result of this analysis. Finally, the small number of participants with key risk factors for LTBI (HIV, immunosuppressed conditions, etc.) and overall small numbers in the completion group limited the power of multivariable analysis.

## Conclusion

Initiation of treatment was the major challenge in our program. Investment in non-employment based screening, social support, and in treatment completion by persons at high risk of developing TB, may improve control efforts in low-incidence settings.

## Competing interests

The authors declare that they have no competing interests.

## Authors’ contributions

JES, LBG, and NDG conceived of the study, and participated in study design and coordination. CP, DB, MAA, and MLMB participated in study design and coordination. NDG and JES performed statistical analysis and drafted the manuscript. TO and JS helped with data analysis and drafting of the manuscript. All authors read and approved the final manuscript.

## Pre-publication history

The pre-publication history for this paper can be accessed here:

http://www.biomedcentral.com/1471-2458/12/468/prepub
